# Potential magnetic drug targeting with magnetite nanoparticles in cancer treatment by enhancer-modifier natural herb and loaded drug

**DOI:** 10.1016/j.heliyon.2024.e32484

**Published:** 2024-06-05

**Authors:** Maria Waqar, Syeda Ammara Batool, Zahida Yaqoob, Jawad Manzur, Mohamed Abbas, Thafasalijyas Vayalpurayil, Muhammad Atiq Ur Rehman

**Affiliations:** a Department of Materials Science & Engineering, Institute of Space Technology Islamabad, 1, Islamabad Highway, Islamabad, 44000, Pakistan; b Centre of Excellence in Biomaterials and Tissue Engineering, Department of Materials Science and Engineering Government College University Lahore, 54000, Pakistan; c Central Labs, King Khalid University, AlQura'a, Abha, P.O. Box 960, Saudi Arabia; d Electrical Engineering Department, College of Engineering, King Khalid University, Abha 61421, Saudi Arabia

**Keywords:** Magnetite nanoparticles, Cancer, Epilim, Moringa, Magnetic targeting

## Abstract

In the present study, we prepared magnetite nanoparticles (MNPs) loaded with natural *Moringa oleifera* (*M. olf*) herb and Epilim (Ep) drug to evaluate the anti-cancerous activity against brain cancer cells. All the samples were prepared via co-precipitation approach modified with different concentrations of *M. olf* and Ep drug at room temperature. The MNPs loaded with drug and natural herb were studied in terms of crystal structure, morphology, colloidal stability, size distribution, and magnetic properties. Field emission scanning electron microscopy (FESEM) images exhibited the morphologies of samples with spherical shape as well as the particles size of 9 nm for MNPs and up to 23 nm for its composites. The results of vibrating sample magnetometer (VSM) indicated the magnetization saturation (Ms) of 42.510 emu/g for MNPs. This value reduced to 16–35 emu/g upon loading MNPs with different concentrations of *M. olf* and Ep. Fourier transform infrared spectroscopy (FTIR) indicated the chemical interaction between the Ep, *M.olf* and MNPs. Brunauer-Emmett-Teller (BET) analysis confirmed the largest surface area for MNPs (422.61 m^2^/g) which gradually reduced on addition of *M. olf* and Ep indicating the successful loading. The zeta potential measurements indicated that the MNPs and MNPs loaded with *M. olf* and Ep are negatively charged and can be dispersed in the suspension. Furthermore, U87 human glioblastoma cell line was used for the *in vitro* cellular studies to determine the efficacy of synthesized MNPs against cancer cells. The results confirmed the anti-proliferative activity of the MNPs loaded with *M. olf* and Ep.

## Graphical abstract

**Figure:**
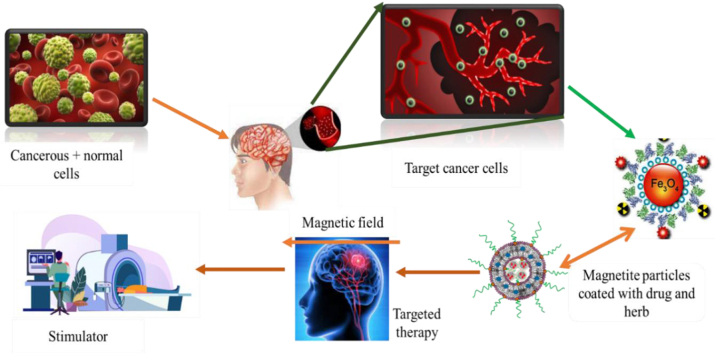

## 1 Introduction

Cancer continues to be one of the most devastating disease due to growing mortality rate. The number of people dying from cancer worldwide is expected to reach 12 million in 2030 [1,2]. Therefore, several courses of action are investigated to cure cancer such as surgery, radiation, and medication. However, these treatments have the disadvantage of causing damage to nearby healthy cells [3]. Plants and their extracts are actively used for medicinal purposes throughout the world. The utilization of medicinal plants is beneficial for formulation since their bioactive components influence many biological signaling pathways. Roughly two third of currently marketed and approved drugs are developed by these natural resources [4]. The green technology explore these natural herbs for cancer therapy [5]. With the rapid advancement of nanotechnology, magnetic nanoparticles (MNPs) have shown a wide range of applications in field of apoptosis, cell separation, and enzyme immobilization [6,7]. The chemotherapeutic drugs can be delivered and released gradually with the use of targeted MNPs to improve bioavailability at the tumor site [8]. MNPs propensity for magnetic field has enabled new breakthroughs in the use of MNPs to deliver drugs attached to these particles in the body by the application of an external magnetic field. Consequently, MNPs offer a facile and efficient method for delivering drugs to the targeted areas in the body without harming the surrounding healthy cells [9].

Commercially available anticancer drugs pose threat to human body. Epilim (Ep) is considered an attractive drug due to its antiepileptic and anticancer activity [10,11]. However, the use of Ep is limited due to the severe cytotoxicity against normal cells [12]. Cancer is a fatal disease and obsoleting the commercial anticancer drugs is not a good approach when we can actually benefit from them. Indeed, their use in sensibly reduced amounts is vital to prevent the cytotoxicity. Hence, the resulting lowered effect of commercial drugs can then be compensated by the incorporation of anticancer herbs. Alternatively, synergistic application of a herbal drug with potential to act against cancerous cells is a promising approach to overcome the toxic effect of Ep [13]. Various nano-carriers such as nanoparticles, nano-capsules, liposomes, and quantum dots have been coupled with potential anticancer herbs to treat cancer. These nano-chemotherapeutic drugs have improved pharmaceutical efficacy, stability, and bioavailability with lower cytotoxic effects [14].

The ayurvedic medicine moringa oleferia *(M. olf)* has high concentrations of antioxidants and bioactive substances which contribute significantly to their effectiveness as anticancer agent [15]. *M. olf* is regarded as one of the significant medicinal plants and a variety of its components is used to address a range of human ailments. The toxicity level of *M. olf* is < 1000 mg/kg but may have toxic effects when taken in large doses [16]. It offers several health advantages, including anti-tumor and anti-bacterial properties [17]. The presence of large amount of aromatic compounds in Glucosinolates and Niazimicin in *M. olf* is effective against cancer cells [18,19]. Table 1 provides a brief overview of work done by various researcher using herbs with or without synthetic drugs for cancer therapy.

**Table 1:** An overview of similar studies for cancer therapy.

Authors	Drug System	Particle size, magnetic behavior	In-vitro or vivo test against Cancer cell	Conclusion	Ref
Navid et al.	MNPs + Curcumin	50 nm 68 emu/g	In vitro cyto-compatibility test against SK-N-MC cell line	The prepared tumor-targeted drug delivery can be used as theranostic agent	[20]
Longzhang et al.	Chitosan coated MNPs + 5-Fluorouracil	20 nm 74 emu/g	In vitro study against SPCA1 cells	Chitosan coated MNPs have excellent potential as novel carriers of 5-Fu for breast cancer chemotherapy.	[21]
Wang et al.	MNPs + Wogonin	5–30 nm	In vitro and In vivo cell studies against Raji cells	Combination of wogonin and magnetic particles is a promising strategy for lymphoma therapy	[22]
Taherian et al.	MNPs + pomegranate peel extract	27.9 nm 30.2 emu/g	In vitro test against NIH/3T3, MBA-MB-231, and 4T1 cells	The novel black pomegranate peel extract loaded with chitosan-coated MNPs has potential for breast cancer therapy	[23]
Raziyeh et al.	MNPs + carbon quantum dots + Doxorubicin	23−75 nm 57.3 emu/g	In vitro test against MCF-7 cancer cells	The synthesized nanocomposite has fluorescence properties that can be used for breast cancer therapy.	[24]
Karunamoorthy et al.	Ag nanoparticles + Moringa	–	cell viability against HeLa cells	M. oleifera has anti-proliferative effect on human cervical carcinoma cells.	[25]

Wang et al. [22] reported the loading of Chinese traditional medicine wogonin with MNPs to evaluate their efficacy in tumor therapy. In this paper, the wogonin was conjugated with MNPs by mechanical adsorption polymerization. The herbal combination with MNPs resulted in enhanced therapeutic efficiency against lymphoma. Another combination of MNPs and herb was explored by Murali et al. [26] for breast cancer therapeutics and imaging application. They investigated the MNPs loaded with curcumin developed by diffusion method. The resultant MNPs demonstrated potent anticancer activity. Similarly, chitosan coated core-shell MNPs were synthesized by Taherian et al. [27] via coprecipitation method. The black pomegranate peel extract was loaded into the MNPs for breast cancer treatment. The *in vitro* cell studies against 4T1 and MDA-MB-231 breast cancer cell lines showed the cytotoxic potential of MNPs proposed in this study. Therefore, the herbal combination of MNPs for cancer treatment is catching the attention of researchers gradually. However, these studies do not compare the effect of these novel combinations to the drugs that are already available and accepted for cancer treatment. Our study also signifies the importance of in-hand solutions and their continuous improvement for better results instead of always experimenting with the new materials which is, of course, imperative for the advancement of knowledge but cannot surpass the importance of accessible drugs. The study also contributes towards the existing knowledge of Ep drug and its efficacy for the cancer treatment.

In the present work, we developed a combination of MNPs incorporating the natural herb *M. olf* using co-precipitation method for the first time according to the best of our knowledge. The objective was to compare the anticancer activity and improve the biocompatibility of this novel combination as compared to Ep drug for brain cancer therapy. All the samples were characterized by Field emission scanning electron microscopy (FESEM), Fourier transform infrared spectroscopy (FTIR), Vibrating sample magnetometry (VSM), X-ray diffraction (XRD), and zeta potential to evaluate their morphological, structural, magnetic, and crystalline properties, respectively. The apoptosis of the proposed *M. olf* -MNPs combination was examined against U87 cell line extracted from glioblastoma which is the most malignant type of brain tumor.

## 2 Materials and methodology

a. Materials

Iron tri-chloride hexa-hydrate (FeCl_3_.6H_2_O; purity>97 %), iron di-chloride tetra-hydrate (FeCl_2_.4H_2_O) analytical grade, sodium hydroxide (NaOH) pellets (∼99 % purity), sodium citrate (Na_3_C_6_H_5_O_7_) (99.5 % purity level), absolute ethanol (C_2_H_5_OH) (purity level >99.8 %) were purchased from Sigma-Aldrich® (Sigma Aldrich, Steinheim, Germany). *M. olf* leaves were purchased from Real Foods (Pvt.) Ltd. Lahore, Pakistan. Ep drug was obtained from Northwest Pharmacy.

b. Synthesis of Fe_3_O_4_ MNPs

Iron oxide (Fe_3_O_4_) MNPs were synthesized via co-precipitation method [28]. In brief, 0.1 M solution of FeCl_3_, 0.2 M of FeCl_2_ and 0.43 M of sodium citrate solutions were prepared in double deionized water (pure water) by stirring for 10 min. Then all solutions were mixed together under constant stirring. After 30min, 3 M NaOH solution was added dropwise in the prepared mixture to maintain the pH 10–12 under constant stirring. After the slurry was ready it was washed with pure water and ethanol until the pH was 7. Next, the precipitates were dried at 60 °C for 24 h under nitrogen environment.

c. Extraction of *M. olf* leaves powder

Fresh leaves of *M. olf* were purchased from Real foods (Pvt.) Ltd. Lahore, Pakistan. The leaves were dried at room temperature, and grinded to a fine powder. To extract 1 g powder, 10 mL ethanol was used. The extraction was done by stirring for 6 h until all the soluble were dissolved into the solvent. After that the mixture was centrifuged for 10 min at 3000 rpm. The extract was washed thrice with ethanol and kept for drying at 50 °C [29]. The extracts of *M. olf* fresh leaves contain eugenol, dibutyl phthalate, 2- chloro-propionic acid and hexadecenoic acid (palmitic acid). Palmitic acid is responsible for in vivo anti-tumor activity in mice [30].

d. *M. olf* and Ep drug Loading in Fe_3_O_4_ MNPs

Four different concentrations of *M. olf* powder and Ep drug were added into the prepared slurry of Fe_3_O_4_ MNPs given in Table 2.

**Table 2:** Concentrations of *M. olf* powder and Ep drug in Fe_3_O_4_ MNPs.

Sample	Composition
S1	Fe_3_O_4_ MNPs +0.6 mL Ep
S2	Fe_3_O_4_ MNPs +2 mL Ep
S3	Fe_3_O_4_ MNPs +0.7 g *M. olf* +0.6 mL Ep
S4	Fe_3_O_4_ MNPs +0.7 g *M. olf* + 2 mL Ep
S5	Fe_3_O_4_ MNPs

## 3 Characterization techniques

The morphology of the samples was examined by field emission scanning electron microscopy (FESEM: Tescan-MIRAIII). Nitrogen adsorption isotherm was used to compute the surface area of Fe_3_O_4_ MNPs and its composites using Brunauer-Emmett-Teller (BET: Gold APP, V-Sorb 2800, China). 20 mg of each sample powder was degassed at 200 °C for 4 h prior to BET analysis. Vibrating sample magnetometer (VSM) was used to determine the magnetization of Fe_3_O_4_ MNPs and its composites. Each sample was placed under the uniform magnetic field which resulted in the magnetic moments. To determine the functional groups in each sample Fourier-transform infrared spectroscopy (FTIR: Nicolet Summit LITE) in transmission mode was done in the range of 4000–500 cm^−1^ with the scan speed of 4 ms^−1^ [31]. X-ray diffraction (XRD: Panalytical-PW3719) analysis of powdered samples was carried out at 2θ range of 30°–70° (stepsize:0.02) using Cu-Kα radiation. Zeta potential (Malven Zetasizer Nanozo90) analysis of each sample was done at 17° angle in a dilute suspension of 0.1 g/L.

a. Cellular studies

Glioma is the most frequent and fatal tumor in the central nervous system; the U87 human glioblastoma cell line is commonly used to study brain cancer, including severe malignant gliomas [32]. U87 cell from human origin were cultured as previously reported [33]. Briefly, 5 × 10^10^ cells per mL were cultured in Dulbecco's modified Eagle's medium (DMEM) enriched with 10 % fetal bovine serum (FBS, heat inactivated), 2 % penicillin, and 2 % streptomycin at 37 °C for 24 h. U87 cell lines were subculture two times in a week at 70–80 % confluency. Later, the cells were rinsed two times a week with Dulbecco's phosphate buffered saline (DPBS). After removing DPBS, cells were incubated for 10 min with the addition of 2 mL of trypsin and 4 mL of DMEM.

To study cell morphology, U87 cell lines were placed in 24-well plates (5 × 10^10^ cells/mL) and incubated for 24 h. Cell lines were centrifuged at 2300 rpm for 5 min. Supernatant was removed and treated with MNPs along with its composites. Later on, samples were incubated for another 24 h and morphology of cells was recorded under an optical microscope.

For cell proliferation studies, the MTT test was employed to evaluate cellular proliferation. Trypan blue was used to count cells using a hemocytometer, and only living cells were counted in order to determine the effect of different concentrations of MNPs and its composites on U87 cell lines. 24 well plates being seeded with 5 × 10^10^ cells/mL were kept in an incubator for 24 h after been diluted with DMEM. Later on, samples containing control, MNPs and its composites were placed in the culture medium to check the effect of prepared MNPs and its composites on the U87 cell lines. For that, the media were extracted, 500 μL of fresh medium supplemented with different concentrations of MNPs, MNPs/Ep, and MNPs/Ep/*M.olf* ranging from 0 mM to 10 mM were added, and the mixture was incubated for 24 h. Following gently pipetting the medium containing the test samples, 500 μL of media containing tetrazolium dye (MTT, 1 mg mL1 final concentration) was added. The media were then incubated for 4 h, and the plates were shaken for a few minutes to homogenize the well contents, and the absorbance was measured at 540 nm by an automatic microplate reader to check the %inhibition of prepared samples. Different concentrations of MNPs and its composites were prepared ranging from 0 mM–10 mM were prepared. 500 μL of each concentration was used to determine the % inhibition. The concentration which provides highest % inhibition with no cytotoxic effect was used to check the anti-proliferative activity of prepared samples against U87 cell lines. Later on, all assays were performed in three replicates.

## 4 Results and discussions

a. Scanning electron microscopy (SEM)

**Fig. 1:**
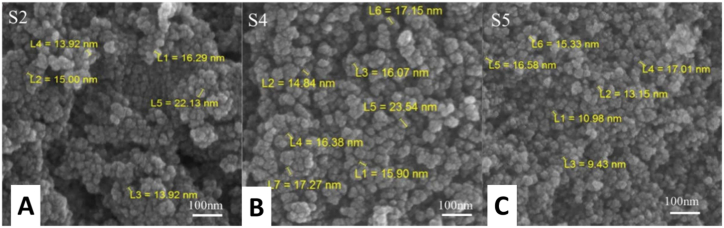
FESEM images of Fe_3_O_4_ MNPs and its composites (S2, S4, and S5) showing the morphology of the synthesized; (A) S2, (B) S4, and (C) S particles.

The size of Fe_3_O_4_ MNPs is the major parameter when they are used for drug delivery systems. The size of these nanoparticles should range up to 10–200 nm to circulate steadily in blood and further accumulate in tumor sites [34]. SEM results are given in Fig. 1 (A, B, and C) which shows the spherical morphology of the S2, S4, and S5 particles. Spherical Fe_3_O_4_ MNPs provides high surface area-to-volume ratio, for drug loading. According to the images the agglomeration is evenly distributed they are not clustered which concludes that there were the formations of the particles. The size of the particles varies with the addition of M. *olf* and Ep drug as given in Table 3, this change in size was due to the surface layer formation. The average particle sizes range between 9 and 23 nm which is ideal for drug delivery system for tumor sites. The size of Fe_3_O_4_ MNPs mostly depends on the synthesis route. The coprecipitation method can yield to more uniform distribution and good magnetic properties of the particles. Sizes of MNPs between 10 and 50 nm are considered to be ideal for cellular studies. Smaller nanoparticles may circulate in the body for longer periods of time and pass through smaller capillaries, which improves the ability to target cancer cells more efficiently. Fe_3_O_4_ nanoparticles ranging from 20 to 50 nm are ideal for drug delivery to cancer cells [35]. The shape of MNPs influences their cellular uptake and distribution within the body. The shape affects how MNPs are dispersed throughout the body, particularly their circulation time in the bloodstream. Spherical particles may have an ideal circulation profile, which is useful for drug delivery [36].

b. Vibrating sample magnetometer (VSM)

**Table 3:** Average size particles of Fe_3_O_4_ MNPs and its composites by FESEM analysis.

Sample	Average Particle Size (nm)
S2	16 ± 0.01
S4	23 ± 0.01
S5	9 ± 0.02

**Fig. 2:**
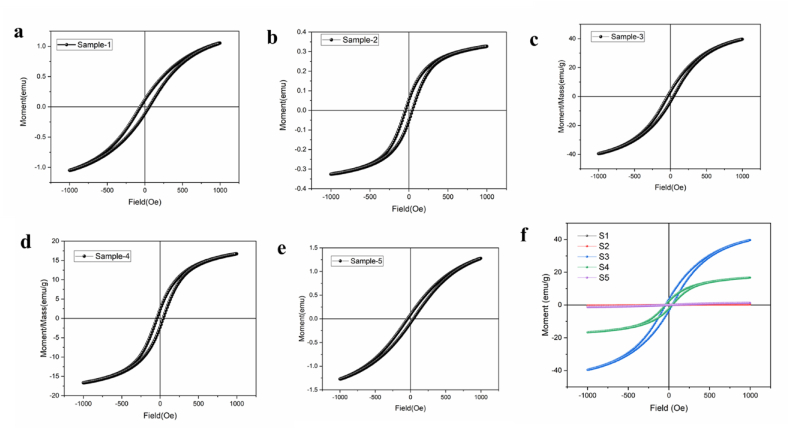
VSM of Fe_3_O_4_ MNPs and its composites (a) MNPs and 0.6 mL Ep (b) MNPs and 2 mL Ep (c) MNPs, 0.7 g M.olf and 0.6 mL Ep (d) MNPs, 0.7 g M.olf and 2 mL Ep (e) MNPs (f) Total magnetic behavior of all samples.

The magnetic properties of each sample were measured by VSM analysis in the field range of −1000 to 1000 Oe, the results were demonstrated in the form of magnetic residual curves shown in Fig. 2(a–f). From hysteresis curves in Fig. 2(a–f), we can acquire the value of saturation magnetization, remanent magnetization, and coercivity field, which is presented in Table 4. The saturation magnetization (M_s_) of Fe_3_O_4_ MNPs and its composites shows the values of 35.007, 10.877, 39.600, 16.690, 42.510 emu/g for S1, S2, S3, S4 and S5 respectively. As it can be clearly observed that the M_s_ value decreases as there was addition of *M. olf* and Ep drug, the main reason behind is the non-magnetic surface layer formation on Fe_3_O_4_ MNPs. The amount of saturated magnetization obtained for Fe_3_O_4_ MNPs (S5) is about 42.510 emu/g which is higher than the reported value 39.5 emu/g [37]. It can be seen that the values of coercivity and remanence magnetization of the samples are very small, indicating the superparamagnetic property of the samples, which is ideal in targeted drug delivery. Since the super paramagnetic property of Fe_3_O_4_ MNPs, according to previous studies, the dimension is reported below 25 nm. The super para-magnetism of the samples synthesized in this study can be further supported by these studies [38,39]. In the absence of residual magnetization, super para-magnetism is important in targeted drug delivery applications. This property prevents MNPs from clustering in blood vessels after the magnetic field is removed. Also, due to their small size, superparamagnetic nanoparticles do not exhibit magnetization unless an external magnetic field is present [40].

c. Fourier transform infrared (FTIR) spectroscopy

**Table 4:** VSM of Fe_3_O_4_ MNPs and its composites.

Sample	Coercivity field (Oe)	Saturation magnetization (M_s)_ (emu/g)	Remanent magnetization (M_r_) (emu/g)
S1	58.519	35.007	3.8387
S2	43.395	10.877	1.7421
S3	40.733	39.600	3.5473
S4	40.288	16.690	2.2386
S5	41.358	42.510	2.9748

**Fig. 3:**
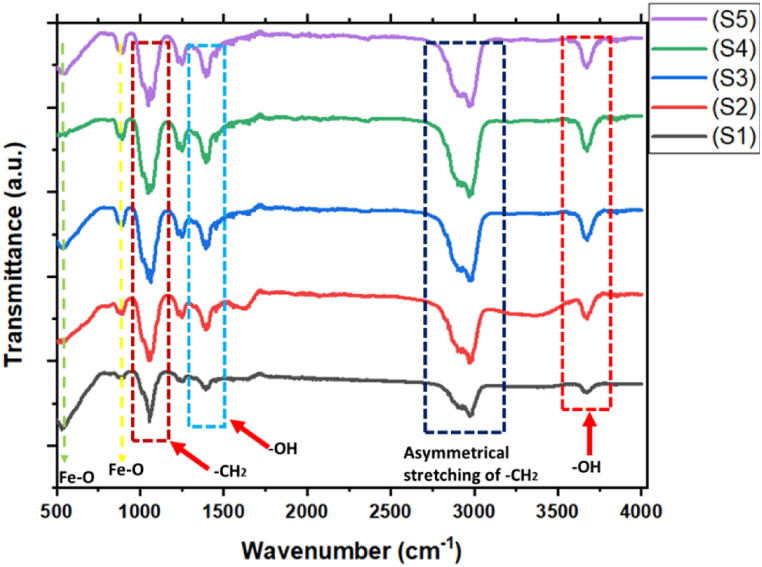
FTIR spectroscopy of Fe_3_O_4_ MNPs and its composites (S1) MNPs and 0.6 mL Ep (S2) MNPs and 2 mL EP (S3) MNPs, 0.7 g *M.olf* and 0.6 mL Ep (S4) MNPs, 0.7 g *M.olf* and 2 mL Ep (S5) MNPs.

To identify the functional groups, structure, and chemical bonds in the Fe_3_O_4_ MNPs and its composites, the FTIR spectroscopy was used. Fig. 3 shows the transmission spectra of each sample. Peaks at 570 and 630 cm^−1^ in the pattern indicate magnetic characteristic related to Fe–O bands in the crystal lattice of Fe_3_O_4_. Peaks at 1629 cm^−1^ and 3435 cm^−1^ are related to the presence of hydroxyl groups related to OH-bending and OH-stretching respectively. There was slight shifting of bands due to loading of M. *olf* and Ep drug. As shown in the FTIR pattern the peaks at 3400 cm^−1^, 1400-1500 cm^−1^ and 1250 cm^−1^ are related to the loading of *M. olf* powder –OH, C=O, and C–O functional group respectively. Due to the band overlap and low band intensity, the low-frequency spectral region <1500 cm^−1^ is related to –CH_2_ stretching which originates mainly from Ep drug.

d. X-ray diffraction analysis

**Fig. 4:**
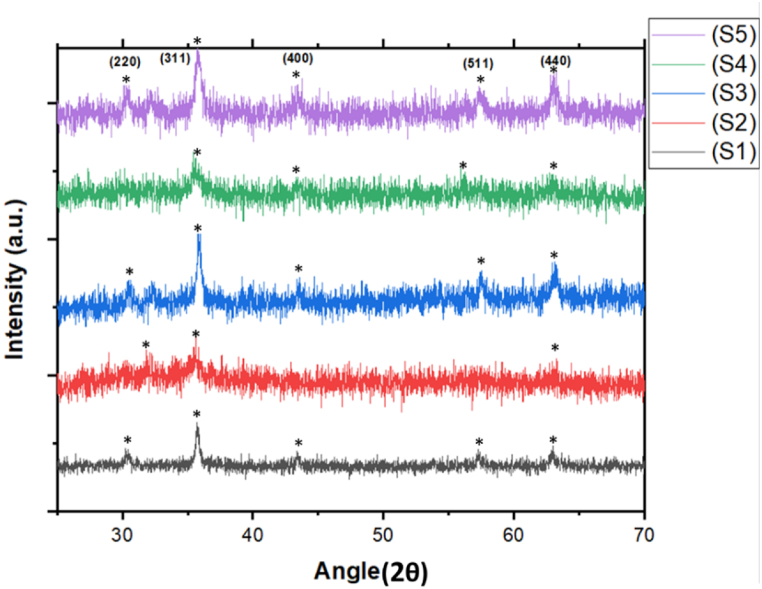
XRD patterns of pure MNPs and its composites loaded with Ep and *M. olf* in different concentrations (S1, S2, S3, S4, and S5).

XRD analysis was done to determine the crystalline nature of each sample. As shown in Fig. 4 peaks match well with the characteristic peaks of spherical structure (JCPDS 21–0139) which shows the crystalline nature of Fe_3_O_4_ MNPs before and after loading of M. *olf* and Ep drug in different concentrations. The strongest peak observed in Fig. 4 at 2θ = 36.7° and 43.2° which corresponds to iron oxide hematite phase [41]. Crystal size is calculated using the Scherer equation (Equation (1)). The FWHM (Full Width at Half Maximum) value is obtained from the results of X-ray diffraction peak fittings using the Gaussian function. The calculation results of crystal size are shown in Table 5 for Fe_3_O_4_ MNPs and its composites.

(Equation 1) $$D=\frac{K.\lambda }{B.\;\cos\;\theta }$$

**Table 5:** Crystalline size and FWHM of S1, S2, S3, S4, and S5.

Sample	Crystallite size (nm)	FWHM
S1	17.7	0.2047
S2	19.2	0.7488
S3	20	0.6140
S4	25.2	0.4093
S5	14.7	0.4093

k is the Scherer constant = 0.9, λ is the Cu wavelength = 0.154056 (Å), B is the FWHM of X-ray diffraction peaks, θ is the Bragg angle.

e. Surface area analysis

**Fig. 5:**
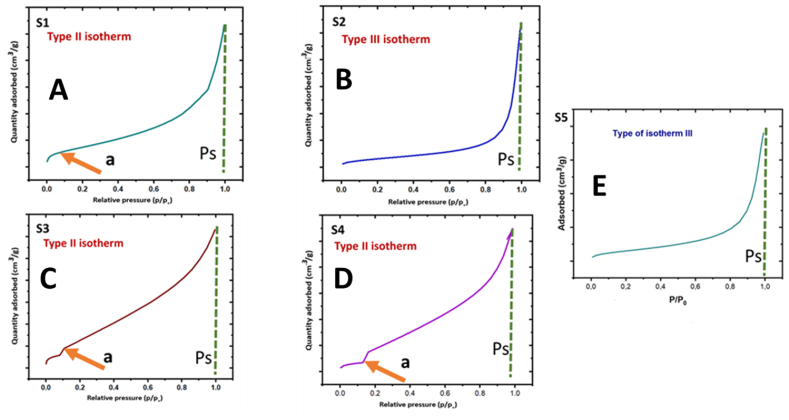
Nitrogen adsorption isotherm of the (A) S1, (B) S2, (C) S3, (D) S4, and (E) S5, particles.

To investigate the surface area of Fe_3_O_4_ MNPs and its composites, BET analysis was conducted with adsorption and desorption isotherms, as shown in Fig. 5 (A – E). The isotherms of S1, S3 and S4 closely match typical type II (Fig. 5 A, C, and D) confirming the multilayer formation due to addition of the Ep drug and *M. olf* in different concentrations. The isotherm curve for S2 and S5 closely matches the type III (Fig. 5 B and E) confirming they are non-porous Fe_3_O_4_ MNPs [42]. The BET analysis results are given in Table 6. The S5 has the largest surface area as compared to the other samples. The small size of nanoparticles provides more surface area and surface energy for interaction with the drugs. S5 has the smallest particle size compared to the others, so these results co-relate with the SEM and XRD analysis. Based on the BET analysis it may be concluded that all samples have good adsorption properties. Fe_3_O_4_ MNPs with large surface areas provide more sites for drug loading, resulting in a higher drug loading capacity per unit mass. In drug delivery applications, the surface area of Fe_3_O_4_ MNPs may affect drug release kinetics. Higher surface area nanoparticles may release drugs faster due to higher surface interactions with the surrounding environment. So having a larger surface area approximately 422.61 m^2^/g is beneficial for us in drug delivery. The usual type of isotherm formed with a non-porous or macro-porous adsorbent is the reversible type II isotherm. The frequency of adsorption increases nearly exponentially when the type II isotherm indicates simultaneous unconstrained monolayer-multilayer adsorption. Type II isotherms can be observed in adsorbents with a wide range of pore sizes, but do not show a saturation limit, indicating that several layers will continue to form indefinitely after the monolayer has been completed. It is common to interpret the arrow point as the stage at which monolayer coverage is complete and multilayer adsorption begins to start.

f. Zeta potential

**Table 6:** Isotherm types and BET surface area of Fe_3_O_4_ MNPs and its composite samples.

Samples	Isotherm type (IUPAC)	Average surface area (m^2^/g)
**S1**	Type II	343.10
**S2**	Type III	140.90
**S3**	Type II	60.10
**S4**	Type II	56.86
**S5**	Type III	422.61

The zeta-potential measurement has been recognized as a significant parameter for providing information about the charge of the synthesized Fe_3_O_4_ MNPs and its composites. Moreover, it also gives information about the stability of the particles in the dispersion which also shows the strength of attraction between the neighboring charged particles. The zeta potential value of MNPs and its composites are given in Table 7, which indicates that the MNPs, MNPs/Ep, and MNPs/Ep/*M. olf* exhibited the zeta potential of −40.1 ± 5 mV, −38.9 ± 3 mV, and −25.6 ± 4 mV, respectively. The reported data suggested that MNPs and their composites are stable in the dispersion. Similar results were reported [43,44]. The test was performed in triplicate and the average values along with the standard deviation.

**Table 7:** Zeta potential of MNPs, MNPs/Ep, and MNPs/Ep/*M. olf*.

Samples	Composition	Zeta Potential (mV)
S1	MNPs	−40.1 ± 5
S2	MNPs + Ep	−38.9 ± 3
S3	MNPs + Ep + *M. olf*	−25.6 ± 4

The surface charge of MNPs affects the interactions with cell membranes, which are usually negatively charged. The surface of negatively charged Fe_3_O_4_ nanoparticles can be modified to enhance biocompatibility and allow for the attachment of drugs. Anticancer drugs like Epilim can be conjugated to the surface of these nanoparticles through chemical reactions [45]. Negatively charged Fe_3_O_4_ nanoparticles are powerful tools in drug targeting for cancer treatment. Their usage involves precise synthesis and surface modification, efficient drug loading, passive and active targeting to cancer cells, and controlled drug release mechanisms. These nanoparticles can deliver therapeutic agents directly to tumor sites, minimizing side effects and enhancing anticancer efficacy [46].

g. Cell culture studies

**Fig. 6:**
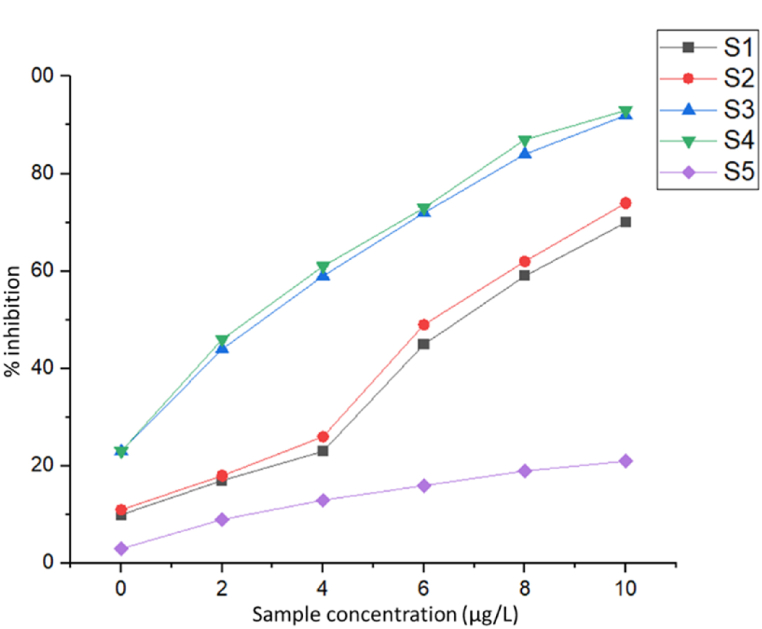
Graph showing the inhibition rate of S1, S2, S3, S4, and S5 against U87 cell lines.

The inhibition of cancer cell growth is critical for therapeutic efficacy, particularly in neuro-oncology, where the suppression of proliferative pathways is essential for patient welfare [30,47,48]. The anti-proliferative activity of compounds S1, S2, S3, S4, and S5 was evaluated using % inhibition and proliferation activity assays (Fig. 6). Different concentrations of the compounds (0 mM, 2 mM, 4 mM, 6 mM, 8 mM, and 10 mM) were applied to U87 glioblastoma cells. The results demonstrated that S1, S2, S3, and S4 were effective cell proliferation suppressors and targeted cancerous cells without significant impact on normal cells. In contrast, S5, containing pure MNPs, had minimal effects on cancer cells and little impact on proliferation. The efficient uptake and bioavailability of S1, S2, S3, and S4 by cancer cells can be attributed to their small size (<20 nm), facilitating their interaction with cellular components.

**Fig. 7:**
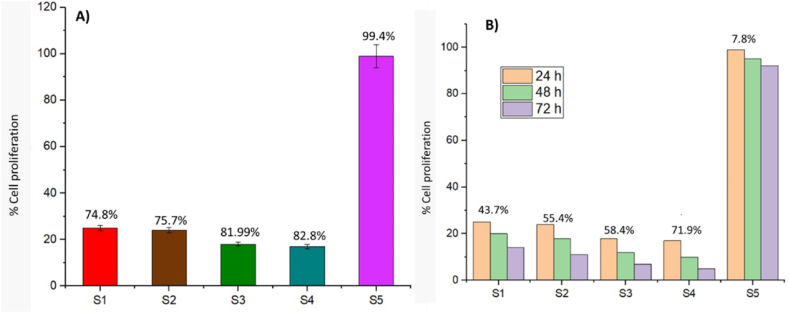
(A) Cell proliferation studies of S1, S2, S3, S4, and S5 against U87 cell lines, and (B) Cell proliferation studies of S1, S2, S3, S4, and S5 against U87 cell lines after 24 h, 48 h, and 72 h.

The highest concentration (10 mM) of S1, S2, S3, and S4 showed significant %inhibition without cytotoxicity to normal cells, indicating selective toxicity toward cancer cells. Conversely, S5 had negligible impact due to its lack of specific targeting mechanisms. Anti-proliferative activity at 24 h, 48 h, and 72 h is shown in Fig. 7A. Notably, S3 and S4 exhibited higher proliferation rates at 24 h, possibly due to the presence of M.olf and Ep, while S1 and S2 showed lower proliferation rates due to lower concentrations of Ep.

The mechanism of action for S1, S2, S3, S4, and S5 primarily involves suppressing cancer cell proliferation by inducing DNA damage through mechanisms such as DNA strand breaks or ion interactions with the cytoskeleton. The degradation of MNPs may also release anticancer ions, causing oxidative stress and altering DNA, ultimately slowing cancer cell growth. This supports existing literature showing the anti-proliferative properties of MNPs and their composites in U87 cell lines and other malignancies [[49], [50], [51], [52]].

The survival rates of U87 cells at varying concentrations and time intervals were shown in Fig. 7B. The results suggest that the anti-proliferative activity of these compounds is both concentration- and time-dependent. Moreover, the study reinforces the importance of dosage control and targeted delivery systems to enhance therapeutic outcomes while minimizing potential side effects on healthy tissues.

Our prepared drug utilizes a targeted delivery system designed to selectively target cancerous cells while sparing normal cells. The mechanism of action involves surface markers or receptors that are overexpressed on cancerous cells, which the drug can specifically bind to. This selective binding allows for the direct delivery of the drug to cancerous cells, limiting exposure to normal cells. Additionally, the drug may be encapsulated in nanoparticles or other carriers that can be programmed to release their payload only upon reaching the targeted cancer cells. Our data demonstrate that this targeted approach effectively delivers the drug to cancerous cells, minimizing the impact on surrounding healthy tissue [53,54].

## 5 Conclusions

MNPs of spherical morphology around 9 nm for S5 in size were successfully synthesized with a gradual increase with the addition of herb and drug which led to a size of approx. 23 nm. The crystallite size determined by XRD were in correspondence with SEM results. S5 was found to be the smallest crystallite size of 14.7 nm. MNPs obtained were super paramagnetic in nature and retained their magnetic behavior even after loading of herb and drug. BET results showed good adsorption properties of all samples. A large surface area of approximately 422.61 m^2^/g for S5 resulted in effective loading of *M. olf* and Ep on MNPs. Studies against U87 brain cancer cells demonstrated that cancer cells could not survive and proliferate in the presence of *M. olf* and Ep loaded MNPs. The highest efficacy was obtained for the samples with higher concentration of *M. olf* i.e. S4. In conclusion, MNPs loaded with *M.olf* in combination with Ep may prove to be effective for potential brain cancer therapy using MDT technique.

## CRediT authorship contribution statement

**Maria Waqar:** Writing – original draft, Validation, Methodology, Investigation, Conceptualization. **Syeda Ammara Batool:** Writing – review & editing, Methodology, Investigation. **Zahida Yaqoob:** Writing – review & editing, Investigation. **Jawad Manzur:** Writing – review & editing, Resources. **Mohamed Abbas:** Writing – review & editing, Resources. **Thafasalijyas Vayalpurayil:** Resources, Investigation, Data curation. **Muhammad Atiq Ur Rehman:** Writing – review & editing, Supervision, Project administration, Investigation, Funding acquisition.

## Declaration of competing interest

The authors declare that they have no known competing financial interests or personal relationships that could have appeared to influence the work reported in this paper.
